# Utilization of Low-Rank Coal and Zn-Bearing Dusts for Preparation of K, Na-Embedded Porous Carbon Material and Metallized Pellets by Synergistic Activation and Reduction Process

**DOI:** 10.3390/ma17235679

**Published:** 2024-11-21

**Authors:** Dingzheng Wang, Deqing Zhu, Jinlin Yang, Shaojian Ma

**Affiliations:** 1 State Key Laboratory of Featured Metal Materials and Life-Cycle Safety for Composite Structures, MOE Key Laboratory of New Processing Technology for Nonferrous Metals and Materials, School of Resources, Environment and Materials, Guangxi University, Nanning 530004, China; dingzheng_wang@163.com (D.W.); msj@gxu.edu.cn (S.M.); 2 School of Minerals Processing and Bioengineering, Central South University, Changsha 410083, China

**Keywords:** Zn-bearing dusts, low-rank coal, porous carbon, reduction, activation, waste disposal

## Abstract

A technology was developed for managing Zn-bearing dust, facilitating the recycling of hazardous solid waste and the production of porous carbon materials. In the one-step process, Zn-bearing dusts were employed not only as raw materials to prepare reduced Zn-bearing dust pellets but also as activators to prepare K, Na-embedded activated carbon. In the process, the Fe, C, Zn, K, and Na in the dusts were rationally utilized. Under optimal conditions, the reduced pellets and porous carbon materials were simultaneously produced and characterized using XRD, SEM/EDS, FTIR, and adsorption of nitrogen techniques. The results indicated that the reduced pellets, with low levels of harmful elements and high iron grade and strength, could be directly used as burden for enhancing blast furnace operation without additional agglomeration. Meanwhile, the K and Na-embedded porous carbon material demonstrated superior SO_2_ and NO adsorption capacities compared to the commercial activated carbon, making it suitable for purifying SO_2_ and NO-bearing flue gas. The hazardous solid wastes were effectively used to treat flue gases through this technology. The mechanism in the synergistic reduction and activation process was elucidated. The coupling effect between the reduction reactions of Fe_2_O_3_, Fe_3_O_4_, FeO, MgFe_2_O_4_, CaFe_2_O_4_, ZnFe_2_O_4_, KFeO_2_, and NaFeO_2_ in the dusts and activation reaction of C in the coal promoted the synchronous reduction and activation process.

## 1. Introduction

Zn-bearing dusts (ZD) are primarily collected in the dedusting systems of blast furnace, converter and electric furnace [1,2,3], and account for 3–4% of crude steel output, and reached about 56–75 million tons in the world in 2023 [4]. The ZD are classified as environmentally hazardous wastes due to their relatively high concentrations of heavy metals (Pb and Zn), which can cause significant environmental pollution without proper management. In the meantime, it is difficult to directly recycle the dusts without removal of Zn from the dusts. The direct reduction technology, due to its high efficiency in removing hazardous metals such as K, Na, Zn, and Pb, has been extensively applied for processing dusts using rotary kiln and rotary hearth furnace methods [5,6]. The carbon-bearing pellets (CBP) made from the dusts are reduced at temperatures ranging from 1100 to 1300 °C during the direct reduction process. The hazardous metal oxides in the dusts can be reduced to their gaseous forms and volatilized into the flue gas, while the iron remains in the pre-reduced pellets. Subsequently, the zinc can be recovered from the flue gas, and the iron-rich pellets can be directly recycled for ironmaking. In direct reduction, the removal efficiency of zinc and lead can exceed 90%, while potassium and sodium can be removed by up to 80% [7,8,9].

Currently, the problems, including the reduction degradation and zinc removal of Zn-bearing dust pellets (ZDP) with high energy consumption in the direct reduction process, constrain its application [10]. When pellets are reduced at high temperatures, the lattice transformation of hematite can cause the pellets to expand in volume, leading to increased fines production, resulting in accretion inside the rotary hearth furnace or rotary kiln and lowering the efficiency of the processes [11,12]. Additionally, using pre-reduced pellets with low compressive strength as charge in the blast furnace necessitates the implementation of certain measures, potentially leading to increased production costs. The reduction temperature for the zinc-bearing dusts is much higher than that of ordinary iron ores for the purpose of hazardous metals removal, which will lead to high energy consumption and poor economic performance [13]. Therefore, it is crucial to develop an effective solution to address these issues. In the coal-based direct reduction process of ZD, metal oxides were reduced by CO and produced CO_2_, which serves as a typical activator for the preparation of activated carbon [14,15]. Moreover, the CO generated during the carbon activation process will promote the reduction of metal oxides in return. Theoretically, it seems that the chain reactions between the reduction of metal oxides in ZD and activation of carbon in the coal can realize the integration of direct reduction of ZD and preparation of coal-based activated carbon. Meanwhile, a catalytic effect on the activation of carbon is likely to be exerted by the zinc, potassium, and sodium in the dusts to some extent [16,17,18]. In previous studies, the zinc-bearing dusts were used as raw material for ironmaking after the removal of zinc [4]. In this study, the zinc-bearing dusts were not only used as raw material for preparation of blast furnace burden but also employed as an activator for the preparation of porous carbon material. Coal is widely used as a precursor for the production of coal-based commercial activated carbon due to its lower cost compared with other precursors, including resin, pitch, shell, and wood. The preparation process of coal-based activated carbon mainly comprises carbonization and activation. Usually, the reaction time of carbonization and activation processes of coal can reach up to 3 h, which will lead to low production efficiency and high energy consumption [19]. Therefore, developing a technology that integrates the carbonization and activation processes is of significant value.

An innovative process combining the direct reduction of metal oxides with the activation of carbon is proposed in this paper to address some limitations of traditional direct reduction methods. ZD from steel plants were made into CBP and utilized for preparation of metallized pellets and porous carbon material simultaneously. The high-value porous carbon material can significantly elevate the economic benefit of the process, while the high-strength reduced pellets can be used directly as a burden to improve blast furnace operations without the need for further agglomeration. In addition, the porous carbon material, which was prepared through a one-step activation process, can be utilized for flue gas desulfurization and denitrification. The adsorption capacity of the porous carbon material for acid gases (SO_2_ and NO) was improved by the presence of alkali metal oxides (K_2_O and Na_2_O) in the material. In this study, the carbonization and activation of coal took place simultaneously in the high-temperature process, shorting the preparation time and elevating the production efficiency compared with the traditional preparation process of coal-based activated carbon. The carbon in the coal functioned as a reducing agent for ZD, whereas the dusts acted as activators for the carbon activation process. The coupling effect between the reduction of ZD and the activation of coal was elucidated. The activation process of the low-rank coal and the phases transformation and microstructure evolution of the ZDP in the process of synergistic activation and reduction were investigated.

## 2. Materials and Methods

### 2.1. Material

In this work, the raw materials collected from an iron and steel plant in Guangdong Province in China were as follows: two kinds of soft coal (SC and YC), one bentonite, and three kinds of ZD, including the dust from converter (DAC), dust from blast furnace (DAB), and environmental dedusting dust of converter (DEC).

The ZD acted as activators for producing the porous carbons (PCs). The main chemical compositions of the ZD are listed in Table 1. It can be observed that the DAC was rich in Fe (56.09%), while the iron content in DAB and DEC was lower than 25%. The carbon-rich DAB could provide a reducing substance for the reduction of metal oxides in ZD. Besides zinc, relatively high contents of potassium (1.25%) and sodium (0.5%) in the blend may constrain the utilization of reduced ZD for ironmaking in a blast furnace [20]. Therefore, the removal rate of potassium and sodium was also investigated in this paper.

Usually, the agglomeration process is needed before the reduced ZD are used for ironmaking. In order to shorten the ironmaking process and improve reduction reactions in ZD, the three kinds of ZD were blended with reductant YC and made into carbon-containing pellets with bentonite as the binder. Bentonite contains 89% montmorillonite and can hold the particles of ZD together and provide cohesion in the green balls [21]. The percentage of particles less than 0.074 mm in the dusts and YC was both higher than 87 wt%, indicating that the size distribution of the materials was suited for pelletizing. Table 2 provides the proximate analysis of YC and the primary chemical compositions of its ash. It was observed that the Vad of YM reached up to 58.2%, which could lead to high porosity of the CBP and promote the diffusion of gases (CO, CO_2_, K, Na, and Zn) in the pellets at high temperature. The SC with a diameter of 10~15 mm was used as a precursor of porous carbons. In the meantime, the SC could provide CO for reduction reactions in the ZDP. The proximate analysis of SC and main chemical compositions of its ash are shown in Table 2. Although the low-rank coal is abundant and cheap, it is classified as a non-renewable fossil fuel. Compared to coal, biomass is cleaner and renewable. Therefore, coal will be replaced by biomass in our further investigation.

### 2.2. Methods

**Figure 1:**
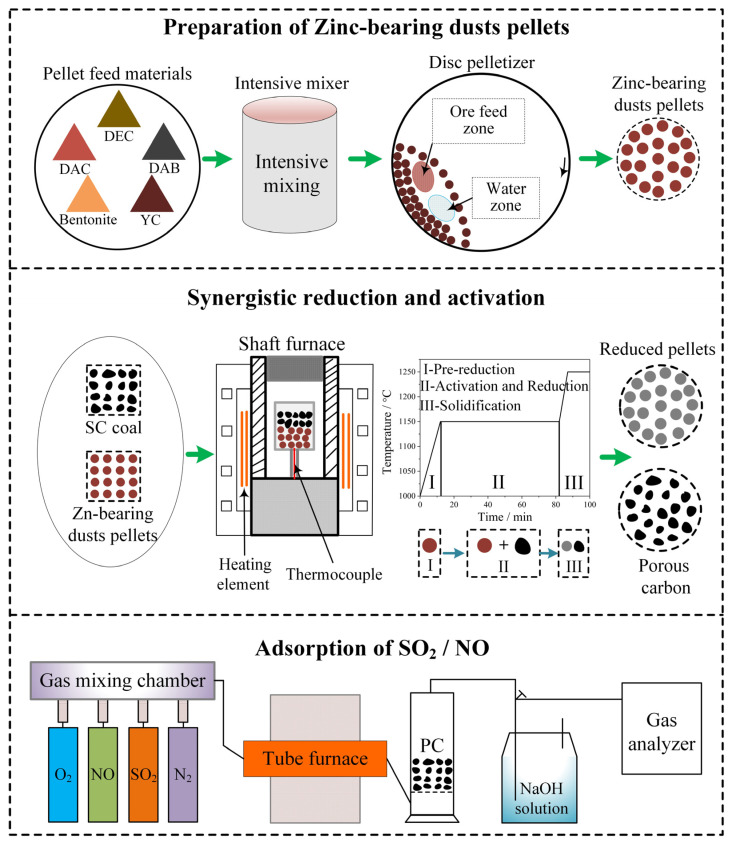
Schematic diagram of the preparation process of metallized pellets and PCs and characterization of SO_2_/NO adsorption capacities of PCs.

This paper primarily investigates the preparation of reduced pellets from ZD and porous carbon materials, the characterization of these reduced pellets and porous carbons, and the demonstration of the mechanism underlying the synergistic reduction of metal oxides and activation of carbon. Figure 1 outlines the process for preparing the porous carbons and reduced pellets. Firstly, carbon-bearing green balls were prepared from a blend of three types of ZD, reductant YC, and bentonite using a disc pelletizer. The mass ratio of YC and swell soil to the blend of ZD were set at 22% and 1%, respectively. Then, the green balls with diameters of 16~18 mm were dried in a vacuum drying oven at 105 °C for 2 h to produce dry pellets. Finally, the dry pellets were reduced, and the SC was activated in a shaft furnace. The experiment conditions were optimized in previous studies.

When the furnace temperature reached 1000 °C, approximately 100 g of dry pellets was introduced into the preheated crucible. The furnace was heated at a rate of 12.5 °C/min, and the pellets underwent a non-isothermal pre-reduction process before the temperature was increased to 1150 °C. Then, some coarse SC was added into the crucible to cover the pellets, and the synergistic reduction of metal oxides and carbonization and activation of carbon began to proceed. The C/O molar ratio, defined as the ratio of fixed carbon in the SC and dry pellets to reducible oxygen in the dry pellets, was set at 1.25 and 2.5. After 70 min of isothermal reaction at 1150 °C, the temperature was raised to 1250 °C in 7 min and then kept for 13 min at this temperature for solidification of the reduced pellets. Finally, the reduced pellets and porous carbon materials were cooled down under the protection of N_2_ and separated through dry magnetic separation.

The adsorption capacities of the porous carbon for SO_2_ and NO were evaluated separately through the apparatus, which is shown in Figure 1. The composition of the mixed gas was set according to the sintering flue gas in BAO steel of China. The inlet gas was adjusted to have an O_2_ volume concentration of 12%, while the mixed gas contained SO_2_ and NO at concentrations of 1800 mg/m^3^ and 360 mg/m^3^, respectively. The mixed gas with a temperature of 80 °C flowed through the porous carbons with a space velocity of 2100 h^−1^. A flue gas analyzer (MGA 5, MRU, Berlin, Germany) was used to monitor and record the concentrations of SO_2_ and NO. The experiment was terminated when the removal efficiency of SO_2_/NO dropped below 20%. The SO_2_/NO adsorption capacity (mg/g) of the porous carbons was calculated by integrating the areas under the adsorption curves along with considering the space velocity and the gas concentration of the mixed gas before adsorption.

The low-temperature N_2_ adsorption–desorption isotherms were determined using a surface area analyzer. The maximum amount of N_2_ adsorbed at a relative pressure of 0.99 defined the total pore volumes of the porous carbons. The surface areas, micropore volumes, and pore size distributions of the porous carbons were measured using the BET method, t-plot method, and DFT analysis, respectively [22]. The iodine number (mg/g carbon) of the porous carbons was measured according to the standard method [23].

The chemical compositions of materials were measured by XRF. To determine the phase compositions of the materials, XRD analysis was performed with Cu Kα1 radiation, a scanning speed of 10°/min, and operating conditions of 40 kV and 150 mA. The microstructure and micro-area composition of the materials were examined by optical microscopy and SEM-EDS. FTIR was used to analyze the functional groups of the porous carbons.

## 3. Results and Discussion

### 3.1. Effects of Reduction Temperature and Time on Properties of the Reduced Pellets and PCs

**Figure 2:**
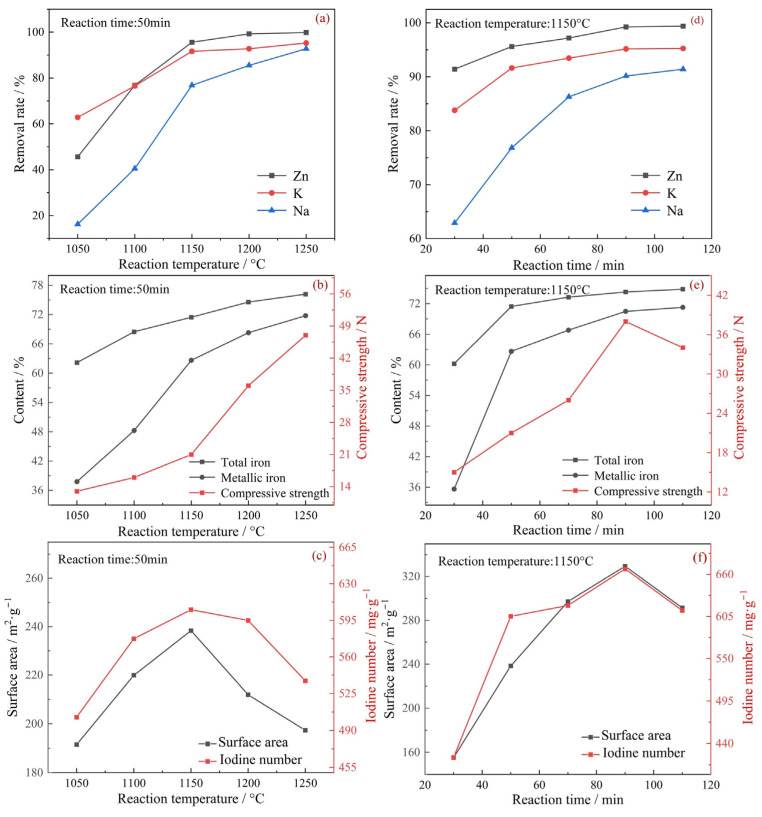
(**a**–**c**) Effects of reduction temperature on properties of the reduced pellets and PCs; (**d**–**f**) Effects of reduction time on properties of the reduced pellets and PCs (C/O = 1.0).

Figure 2 illustrates the impact of reduction temperature and duration on the properties of the reduced pellets and PCs. Figure 2a–c shows that as the reaction temperature rose from 1050 °C to 1250 °C, the iron grade and metallization degree of reduced pellets and the removal rate of zinc, potassium, and sodium continuously increased. This indicated that higher temperatures were advantageous for the reduction of ZD. However, when the reaction temperature exceeded 1150 °C, both the surface area and iodine number of the porous carbon materials decreased, suggesting that excessive temperatures could damage the microstructure of the carbon material.

From Figure 2d–f, the increase in the removal rate of harmful elements and iron reduction degree leveled off gradually when the reduction time reached 90 min. At a reaction temperature of 1150 °C for 90 min with a C/O molar ratio of 1.0, both the porous carbon material, which exhibited a surface area of 329.55 m^2^/g and an iodine number of 667.28 mg/g, and the reduced ZDP, with an iron grade of 74.29% and an iron metallization degree of 94.88%, were obtained. Additionally, the removal rates for zinc, potassium, and sodium reached 99.24%, 95.17%, and 90.15%, respectively. However, the reduced pellets had low compressive strength and could not be used directly as burden for the blast furnace. Therefore, further optimization of the process is required.

### 3.2. Effects of Pre-Reduction on Properties of the Reduced Pellets and PCs

**Figure 3:**
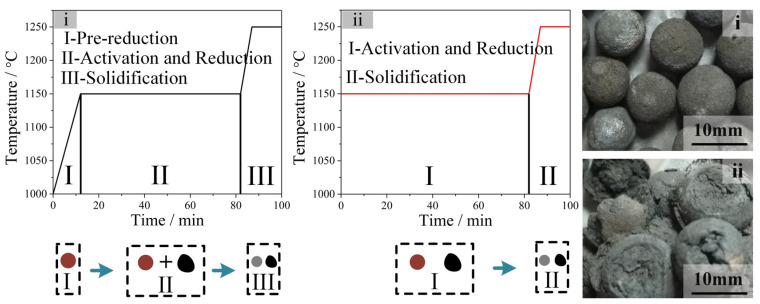
Effect of pre-reduction on morphology of reduced pellets. (**i**) Pre-reduced for 12 min; (**ii**) without pre-reduction.

Figure 3 and Table 3 illustrate the impact of the pre-reduction process on the properties of reduced ZDP. As shown in Figure 3i, the diameter of the pellets reduced from 18 mm to 10 mm following the adaptation of the pre-reduction process. However, the reduced pellets are severe in reduction swelling and reduction degradation when the dry pellets and coal directly progressed to synergistic reduction and activation stage. During the initial stage of reduction, the Fe_2_O_3_ in the pellets can be reduced to Fe_3_O_4_, and the lattice transformation can lead to a volumetric expansion of the pellets [24]. An excessive lattice transformation rate may cause a serious reduction swelling of pellets and bring about a greater generation of irrecoverable cracks. In the pre-reduction process, the relatively low reduction temperature and C/O molar ratio could decrease the reduction rate of hematite and thus prevent the formation of cracks in the pellets.

It can be observed from Table 3 that the reduced pellet (ii) could not be directly used as a burden for blast furnaces due to the low CCS. Pellets (i) and (iii), which possess high CCS, iron grade, and metallization degree, along with low levels of hazardous metals, can be used directly as charge to enhance blast furnace operation without needing further agglomeration.

**Figure 4:**
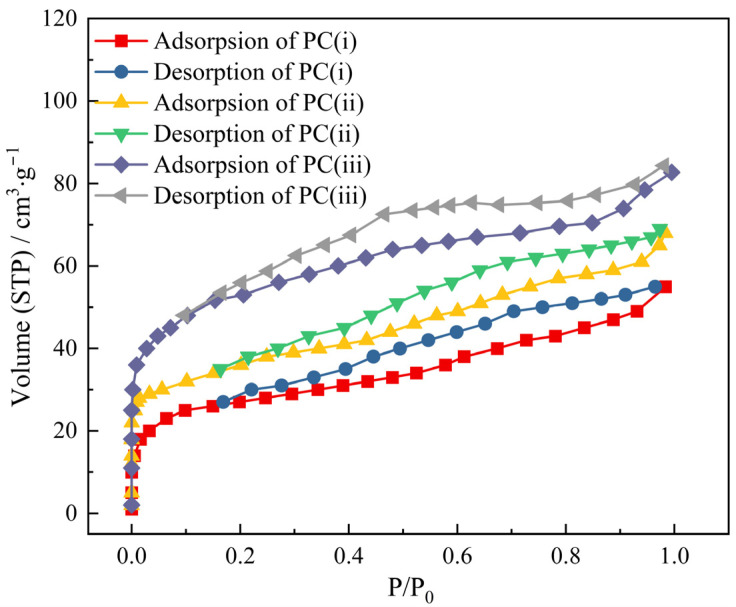
The N_2_ adsorption–desorption isotherms of PC (i)~PC (iii).

Table 4 and Figure 4 presents the impact of the pre-reduction process on the properties of porous carbons. Part of the metal oxides was reduced by the carbon in the pellets in the pre-reduction process, and the generated CO_2_ would flow out of the shaft furnace when the SC coal was added into the furnace. The surface area and iodine number of PC (i) were relatively low due to the absence of activating substance in the activation process. Increasing the mass ratio of zinc-bearing pellets to SC resulted in the formation of PC (iii), which had a surface area of 300.65 m^2^/g and an iodine number of 655.23 mg/g.

### 3.3. Characterization of the Reduced Pellets and Porous Carbon Materials

**Figure 5:**
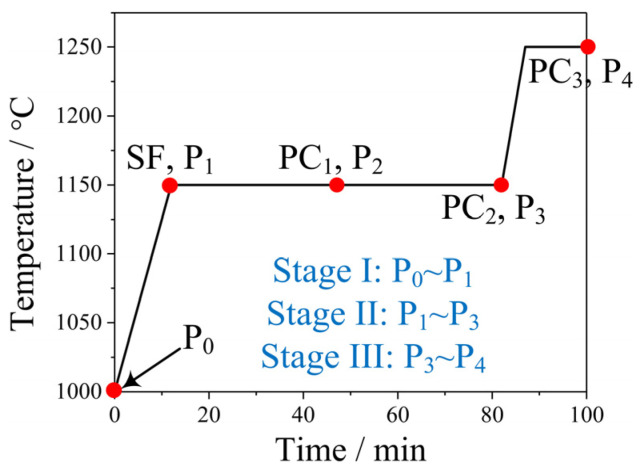
Preparation condition of the PCs and metallized pellets. (C/O = 0.5).

The preparation conditions of the porous carbons and metallized pellets are shown in Figure 5.

The porous carbons (PC_1_~PC_3_), dry pellets (P_0_) before reduction, and reduced pellets (P_1_~P_4_) were fully characterized to investigate the reaction process and reveal mechanism of the synergistic reduction of metal oxides and activation of carbon.

#### 3.3.1. Zn-Bearing Dust Pellets

Table 5 shows the properties of reduced pellets at different stages of reduction. In the pre-reduction process (stage I), significant portions of potassium and sodium were removed from the pellets and the metallization degree of the pellets increased from 2.72% to 44.31%. However, the CCS of the reduced pellets dropped from 156 Newtons per dry pellet to 45 Newtons per pellet due to the increase in the porosity of pellets resulting from the removal of volatile component. The contents of zinc, potassium, and sodium in P_3_ were both lower than 0.05%, indicating that most hazardous metals were removed from the pellets at the end of the synergistic activation and reduction stage (stage II). Although the metallization degree of P_3_ reached 94.55%, the CCS of P_3_ was only 223 Newtons per pellet, which could not meet the requirement for blast furnace operation. The CCS of reduced pellets increased sharply, and the contents of hazardous metals further declined in the high-temperature solidification stage (stage III). Finally, the P_4_ with high content and metallization degree of iron, low content of hazardous metals, and high CCS could be directly recycled as a charge to enhance blast furnace operation.

**Figure 6:**
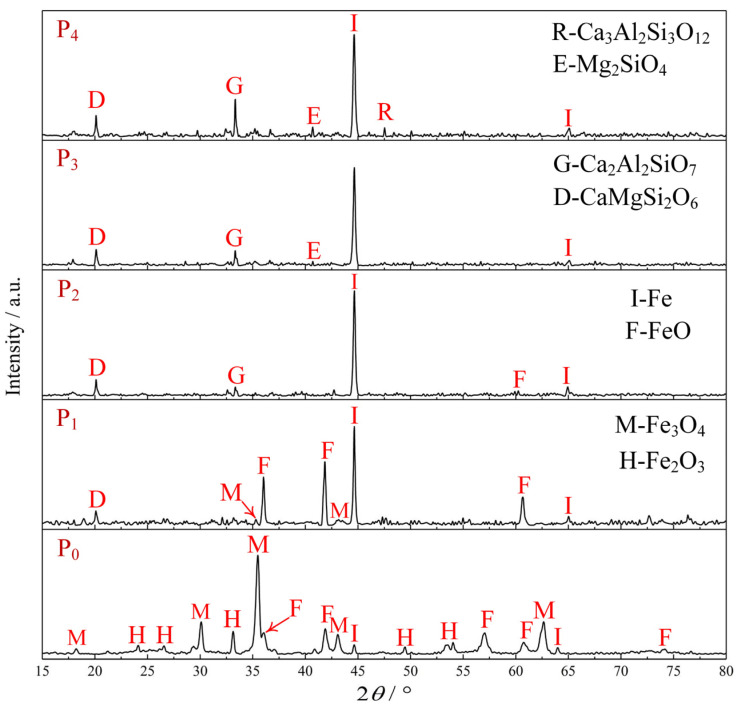
XRD patterns of the ZDP before and after reduction.

Figure 6 illustrates the phases transformation of the ZDP during the reduction process. The diffraction peaks of P_0_ indicate that the dry pellets before reduction were primarily composed of iron-bearing phases, including Fe (metallic iron), FeO (wustite), Fe_3_O_4_ (magnetite), and Fe_2_O_3_ (hematite). Phases not detected in the XRD analysis were examined using SEM/EDS analysis.

Figure 6 (P_1_) shows that the diffraction peaks of Fe_2_O_3_ were negligible, while the intensities of the diffraction peaks for FeO and Fe became stronger. This indicates that most of the hematite and magnetite had been reduced to wustite and metallic iron in the pre-reduction stage. From Figure 6 (P_2_ and P_3_), most of the iron-bearing phases were reduced to metallic iron after stage II. The gangue phases, which included CaMgSi_2_O_6_ (diopside), Ca_2_Al_2_SiO_7_ (gehlenite), and Mg_2_SiO_4_ (forsterite), were gradually generated as the reaction progress and formation of Ca_3_Al_2_Si_3_O_12_ (grossular) required the higher temperature.

**Figure 7:**
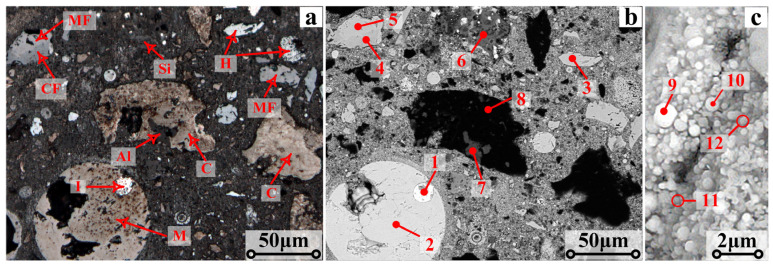
The optical micrograph (**a**) and electronic micrograph (**b**,**c**) of the pellets before reduction. (I—Fe-metallic iron, H—Fe_2_O_3_-hematite, M—Fe_3_O_4_-magnetite, CF—CaFe_2_O_4_-calcium ferrite, MF—MgFe_2_O_4_-magnesioferrite, Al—Al_2_O_3_-alumina, Si—SiO_2_-silica, C—carbon).

Figure 7a presents the optical micrograph of P_0_ and the electronic micrographs in Figure 7b,c. Table 6 provides the elemental compositions of the micro-areas shown in Figure 7. From point 1 in Figure 7b, a small amount of metallic iron grains existed in the ZD before reduction. The spherical particle (point 2) with a size of 80 microns consisted of Fe_3_O_4_ (magnetite).

It was observed that part of the Fe_2_O_3_ (hematite) (point 3) was presented as agglomerates with a particle size ranging from 10 to 25 microns, while other hematite particles with an average particle size of less than 2 microns (point 10) were distributed around the agglomerates. The calcium, magnesium, silicon, and aluminum existed in CaFe_2_O_4_ (calcium ferrite), MgFe_2_O_4_ (magnesioferrite), SiO_2_ (silica), and Al_2_O_3_ (alumina), respectively (points 4~7). The black regions in Figure 7b consisted of carbon, which can generate CO that subsequently reacts with the metal oxides in the pellets. The zinc existed in spherical ZnFe_2_O_4_ (zinc ferrite) grains with a size of 1 micron (point 9). From points 11 and 12 in Figure 7c, the atomic contents of sodium and potassium were only 5.6% and 6.4%, respectively, indicating that these particles consisted of the solid solution of NaFeO_2_/KFeO_2_ and Fe_2_O_3_. Figure 7 illustrates the microstructure and phase transformation of the reduced pellets in the reduction process. The elementary compositions of micro-areas shown in Figure 7 are detailed in Table 7.

**Figure 8:**
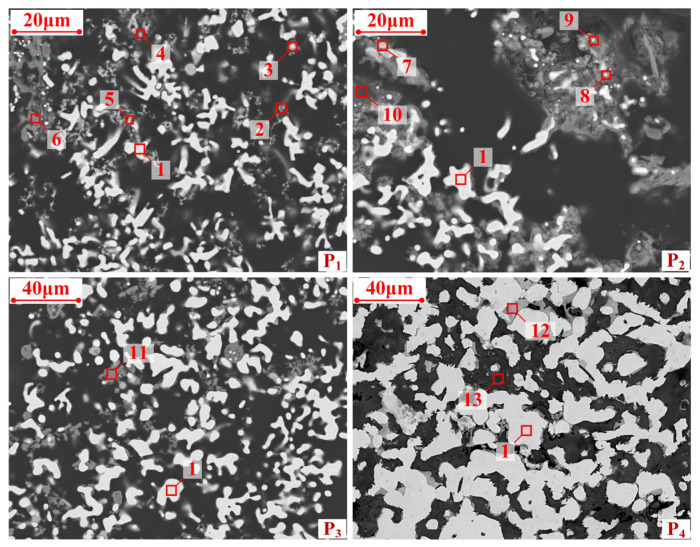
SEM images of the reduced pellets.

From Figure 8 (P_1_), certain amounts of metallic iron (point 1) and wustite (point 2) could be observed in the pre-reduced pellets. The atomic content of zinc in point 3 was found to be 10.3%, which was lower than that of pure ZnFe_2_O_4_, indicating that part of the zinc ferrite was reduced to metallic iron and gaseous zinc in the pre-reduction stage. Similarly, it can be seen from points 4 and 5 that parts of the potassium iron oxide and sodium iron oxide were reduced in stage I. The porosity of the pellets increased due to the removal of volatile components from the pellets, leading to the strength degradation of the pellets.

From Figure 8 (P_2_), most of the fine-grained wustite vanished from the pellets, while zinc ferrite, potassium iron oxide, and sodium iron oxide (point 7, 8 and 9) were still present in the reduced pellets. As shown in Figure 8 (P_3_), most of the harmful elements had been removed, and there is a noticeable increase in the size of the metallic iron grains after stage II. However, the CCS of the P_3_ was still low (223 Newtons per pellets) due to the dispersion state of metallic iron grains. After the solidification stage, the phases in P_4_ were interconnected by metallic iron and magnesioferrite (point 12). With the increase in metallic iron grains, the CCS of the reduced pellets rose from 223 to 905 Newtons per pellet. Reduced pellets with good strength can be used directly as charge to enhance blast furnace operation, without the need for additional agglomeration. The SEM/EDS analyses were consistent with the results of XRD analysis.

#### 3.3.2. Porous Carbon Materials

**Figure 9:**
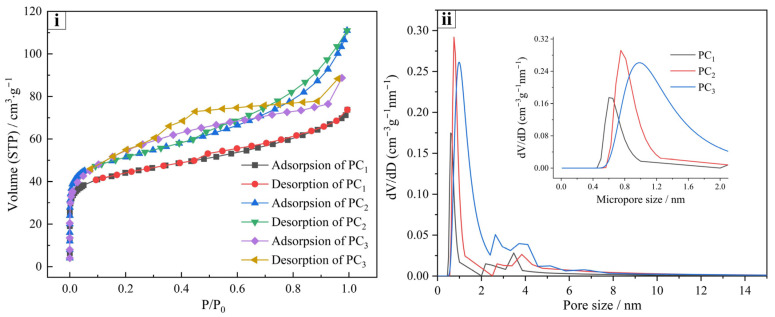
(**i**) N_2_ adsorption–desorption isotherms; (**ii**) Pore size distributions of PCs.

Figure 9i illustrates nitrogen adsorption–desorption isotherms of PCs at 77 K. The dramatic increase in the adsorption curves at a relative pressure of P/P_0_ < 0.1 indicated that the carbon materials have a high content of micropores, and the micropore contents of PC_1_ were lower than those of PC_2_ and PC_3_. The hysteresis loop in the range of 0.3 < P/P_0_ < 0.95 confirmed the presence of certain amounts of mesopores in the carbon materials [25], and the hysteresis loop of PC_3_ possessed a larger area due to the relatively high proportion of mesopores.

According to the IUPAC classification [26,27], the adsorption–desorption isotherms fall under a combination of type I and type IV, indicating a structure that includes both micropores and mesopores. The pore size distribution and surface areas of PCs are shown in Figure 9ii and Table 8. PC_1_ exhibited a surface area of 218.9 m^2^/g, a total pore volume of 0.191 mL/g, and a micropore content of 52.37%. Figure 9 shows that increasing the activation time significantly elevated the surface area and pore volume of the carbon material. The excessive temperature in stage III caused a small portion of the microporous structure of the carbon material to break down. Ultimately, PC_3_ demonstrated a surface area of 300.7 m^2^/g, a total pore volume of 0.264 mL/g, and a micropore content of 48.21%. Although the surface area of P_3_ was lower than that of activated carbons in other studies [28,29], the porous carbon produced as a byproduct of the process can enhance the economic benefit and energy efficiency of the ZD disposal process.

**Figure 10:**
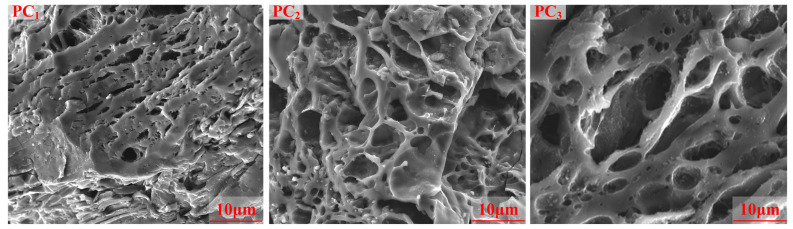
SEM images of PCs.

Figure 10 illustrates the microstructure transformation of porous carbon material during the reduction and activation processes. It can be observed that the surface of PC_2_ presented an apparent microporous structure, while the porosity of PC_1_ was significantly less than that of PC_2_. Pores formed continuously as tarry substances were removed and carbon underwent gasification in stage II. However, some micropores collapsed and more mesopores were formed at high temperatures in stage III. The scanning electron microscopy analysis aligned with the findings from the N_2_ adsorption–desorption analysis.

**Figure 11:**
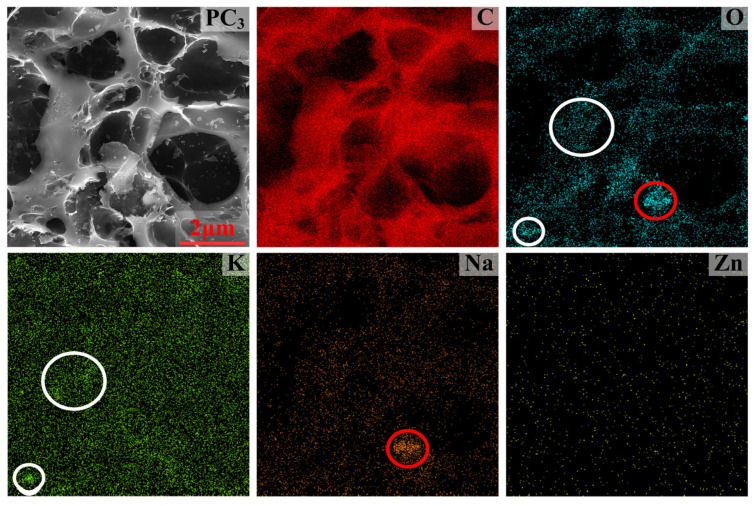
Elements distribution in PC_3_.

The main elements distribution in PC_3_ is shown in Figure 11. It can be observed that potassium and sodium were adsorbed onto the carbon skeleton, with their positional distribution aligning with that of oxygen, indicating that the K and Na elements in the porous carbon material were present as potassium oxide and sodium oxide, respectively. KFeO_2_ and NaFeO_2_ in the dust can be reduced to gaseous metal potassium and sodium, which are adsorbed by the porous carbon material. The contents of K_2_O, Na_2_O, and ZnO in PC_3_ were found at 0.76%, 0.47%, and 0.03%, respectively.

**Figure 12:**
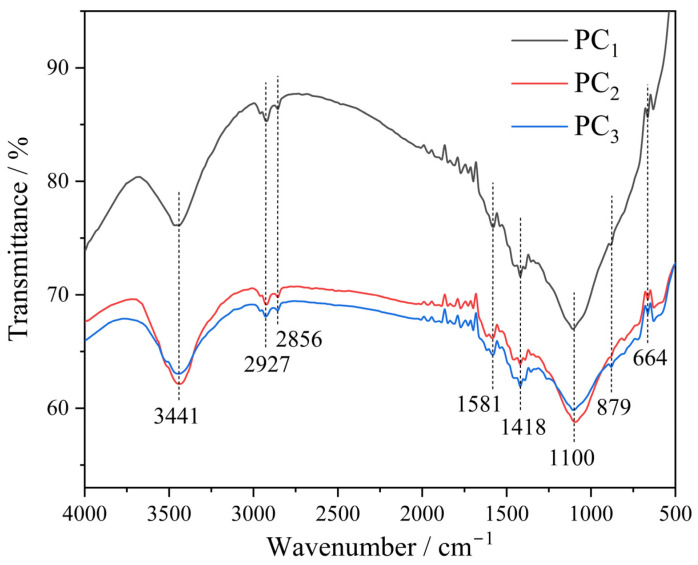
Fourier transform infrared spectroscopy (FTIR) spectra of PCs.

To investigate the functional groups on the surface of the carbon materials, the infrared spectra of PC_1_~PC_3_ were analyzed. The results are presented in Figure 12.

The three spectra showed a similar shape, while the transmittance of PC_1_ was obviously higher than that of PC_2_ and PC_3_, which was probably due to the incomplete activation of PC_1_. The strong and broad bands observed at 3441 cm^−1^ were attributed to the stretching vibration of the hydroxyl functional group (O-H) [30,31]. The bands at 2927 cm^−1^ and its shoulder at 2856 cm^−1^ were ascribed to the stretching and bending vibration of C–H bands, respectively [32]. The bands at 1581 cm^−1^ can be assigned to the stretching vibrations of C=C [33]. The bands at 1418 cm^−1^ were related to some oxygen-bearing groups like ketones and carboxyl [34]. The bands at 1100 cm^−1^ were attributed to C–O–C stretching, while the bands at 879 and 664 cm^−1^ were related to out-of-plane bending in benzene derivatives [33].

In the steel manufacturing process, approximately 70% of SO_2_ and 40% of NOx are produced during the sintering process [35]. Therefore, removing SO_2_ and NOx from the sintering flue gas is essential. Currently, many steel plants have widely adopted activated carbon adsorption methods to purify the sintering flue gas [36].

**Figure 13:**
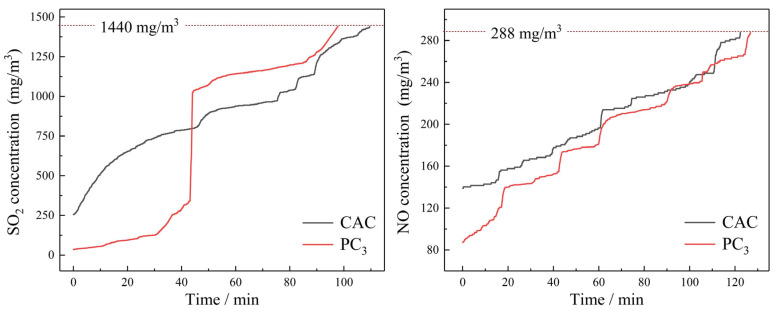
The SO_2_/NO breakthrough curves of the carbon materials at 80 °C.

Figure 13 shows the desulfurization and denitration performances of PC_3_ and the commercial activated carbon (CAC), which is applied to the sintering flue gas purifying process in Bao steel of China. The adsorption capacities for sulfur dioxide were 5.91 mg/g for PC_3_ and 5.48 mg/g for CAC. The adsorption capacities for nitric oxide were 1.19 mg/g for PC_3_ and 1.07 mg/g for CAC. The removal rates of SO_2_ and NO could reach 98% and 62%, respectively, which were higher than those in some previous studies [37,38,39]. Despite PC_3_ having a relatively low surface area [40], the presence of alkaline oxides (K_2_O and Na_2_O) and functional groups enhanced its adsorption of acid gases (SO_2_ and NO). PC_3_ demonstrated higher desulfurization and denitration efficiency compared to CAC, indicating that PC_3_ is more effective at disposing SO_2_ and NO-containing flue gas.

### 3.4. Mechanism of the Synergistic Reduction and Activation

**Figure 14:**
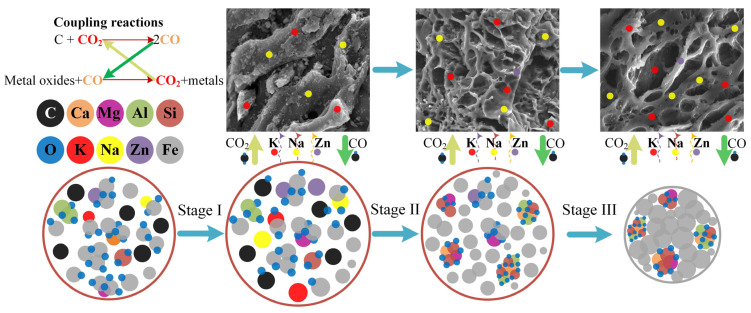
Schematic diagram of the mechanism of synergistic reduction and activation.

Figure 14 illustrates the mechanism of synergistic reduction and activation, with the chemical reactions (1)~(12) involved listed in Table 9.

**Figure 15:**
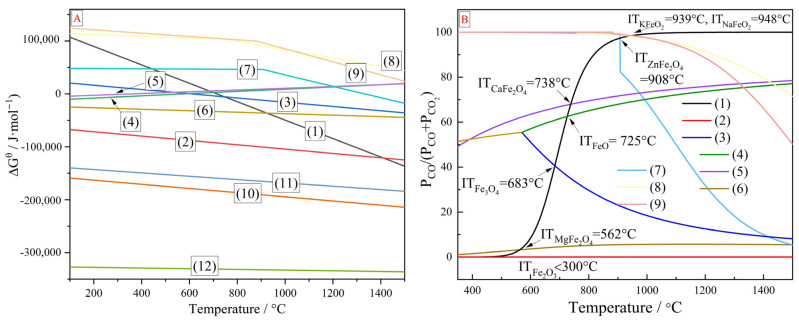
(**A**) Standard Gibbs free energy ΔG^θ^ vs. reaction temperature for the reactions (1)~(12) in Table 9; (**B**) Equilibrium composition of gas phases vs. reaction temperature for the reactions (1)~(9) in Table 9 (IT-initial reaction temperature).

The thermodynamic analysis of these reactions was carried out through Factsage7.1 to investigate their initial reaction temperatures, and the results are presented in Figure 15. Figure 15B shows that the initial reduction temperatures for iron oxides, including hematite, magnetite, wustite, calcium ferrite, and magnesioferrite, were all below 750 °C. In contrast, the initial reduction temperatures for zinc ferrite, potassium iron oxide, and sodium iron oxide were significantly higher, at 908 °C, 939 °C, and 948 °C, respectively. The operation temperatures in this paper were 1150~1250 °C, which were higher than the initial reaction temperatures of the chemical reactions in Table 9. From reactions (2)~(9) in Table 9, metal oxides in ZDP could be reduced by CO to produce CO_2_. In the meantime, the carbon in the coal could be activated by CO_2_ generated from these reduction reactions, and the CO generated from the activation reaction (reaction (1) in Table 9) could facilitate the reduction reactions in return. Therefore, the reduction of metal oxides in ZDP and the activation of carbon in coal were mutually enhanced through these coupling reactions. In addition, the carbon cycle between the coal and dusts could decrease the carbon emission. The in-depth analysis of the carbon cycle in this process and utilization of cleaner materials like biomass will be studied in our further investigation.

It can be seen from Figure 14 that the carbon in the pellets reacted with oxygen and existed in the pellets to form carbon monoxide at the beginning of the pre-reduction process (stage I). Subsequently, the carbon monoxide reacted with nearby metal oxides to produce carbon dioxide. This carbon dioxide then reacted with carbon to generate a reducing gas via the Boudouard reaction [41,42]. These coupling reactions only occurred inside the pellets in stage I. Most of the Fe_2_O_3_ and Fe_3_O_4_ were reduced to FeO, leading to volume expansion of the pellets in stage I. The porosity of the pellets increased with the removal of volatile components and volume expansion, which was beneficial for the diffusion of the gases (CO, CO_2_, Zn, K, and Na). After stage I, the SC coal was added into the furnace, and the coupling reactions were performed between the coal and ZDP. In stage II, the CO produced from the coal activation reaction reduced most of the iron-bearing phases. The strength of the pellets remained low due to the dispersed distribution of metallic iron grains. The vast majority of Zn, K, and Na were removed from the pellets, and considerable amounts of the K and Na, which have a catalytic effect on carbon activation, were absorbed by the porous carbon in the synergistic reduction and activation stage [16,17]. The coal was transferred into the porous carbon material with abundant functional groups and micropores after stage II. In the solidification stage (stage III), almost all the reducible metal oxides had been reduced, and the absence of an activator quenched the chain reactions. Under high-temperature conditions, the metallic iron grains bonded with each other, significantly increasing the strength of the reduced pellets. Finally, the coal was activated by the ZD and transferred into porous carbon material, which can be used to purify the sintering flue gas, while the ZDP were reduced to metallized pellets, which can be directly used as burden to enhance blast furnace operation.

## 4. Conclusions

A process for utilization of ZD and low-rank coal was developed, through which not only the dusts were reduced to the high-quality blast furnace burden but also a low-rank could be transferred into high-value porous carbon material simultaneously. The mechanism of the process of synergistic reduction of metal oxides and activation of carbon was clarified. The conclusions can be drawn as follows:

(1) The reduced zinc-bearing dust pellets with CCS of 905 Newtons per pellet, iron grade of 73.59%, iron metallization degree of 97.03%, and low content of hazardous metals (0.015% Zn, 0.017% K, and 0.039% Na) could be directly used as burden for improving blast furnace operations without further agglomeration.
(2) Although the surface area of the porous carbon was not high, it possessed better SO_2_ and NO adsorption capacities compared with the commercial activated carbon due to abundant functional groups and considerable amounts of alkaline oxides (0.76% K_2_O and 0.47% Na_2_O) in the material.
(3) In the synergistic reduction and activation process, the Fe_2_O_3_, Fe_3_O_4_, FeO, ZnFe_2_O_4_, MgFe_2_O_4_, CaFe_2_O_4_, KFeO_2_, and NaFeO_2_ in Zn-bearing pellets could be reduced by the CO, which was generated from the activation reaction of coal, while the coal was activated by the CO_2_ generated from the reduction reactions. The synchronous reduction and activation processes were promoted by the coupling reactions between the Zn-bearing dust pellets and coal.

## Figures and Tables

**Table 1:** Main chemical compositions of ZD (wt%).

Item	Fe_total_	FeO	MFe	Zn	K	Na
DAC	56.09	26.55	1.72	3.15	0.25	0.18
DAB	24.07	13.30	0.69	7.82	3.13	0.96
DEC	19.81	14.87	0.09	1.38	1.29	1.56
Blend	44.07	21.71	1.2	4.63	1.25	0.50
**C**	**SiO_2_**	**Al_2_O_3_**	**CaO**	**MgO**	**LOI^a^**	**Adding ratio**
-	1.55	0.25	6.24	1.87	3.05	63
20.43	10.00	1.10	4.83	1.48	24.44	33
-	6.17	3.58	23.82	5.55	16.60	4
6.74	4.53	0.67	6.47	1.90	10.65	100

LOI^a^—loss on ignition.

**Table 2:** The proximate analysis of coals and chemical compositions of their ash (wt%).

Coal Types	Proximate Analysis				Main Chemical Compositions of Ash				
	M_ad_	FC_ad_	V_ad_	A_ad_	Fe_total_	CaO	Al_2_O_3_	SiO_2_	MgO
YC	5.6	31.4	58.2	4.7	4.5	28.2	20.7	19.5	21.9
SC	0.1	51.4	29.4	6.1	11.8	25.0	8.0	27.6	16.3

M_ad_: Moisture, FC_ad_: fixed carbon, V_ad_: volatile matter, A_ad_: ash.

**Table 3:** Effects of pre-reduction on the properties of reduced pellets.

Reduced Pellets	C/O	CCS/N per Pellet	Metallization Degree/%	Fe_total_ /%	Zn (%)	K (%)	Na (%)
Pellet (i)	1.0	856	97.83	74.27	0.010	0.007	0.023
Pellet (ii)	1.0	24	97.52	73.93	0.008	0.006	0.021
Pellet (iii)	0.5	905	97.03	73.59	0.015	0.017	0.039

Pellet (i) and pellet (iii): pre-reduced for 12 min; pellet (ii): without pre-reduction; CCS: cold compressive strength.

**Table 4:** Effects of pre-reduction on properties of the porous carbons.

PCs	C/O	Surface Area/m^2^·g^−1^	Iodine Number/mg·g^−1^	Burn-Off/%
PC (i)	1.0	118.20	299.76	49.84
PC (ii)	1.0	134.42	601.35	55.31
PC (iii)	0.5	300.65	655.23	53.24

PC (i) and PC (iii): pre-reduced for 12 min; PC (ii): without pre-reduction.

**Table 5:** The properties of reduced pellets (C/O = 0.5).

Reduced Pellets	CCS/N per Pellet	Metallization Degree/%	Fe_total_ /%	Zn (%)	K (%)	Na (%)
P_0_	156	2.72	35.83	3.76	1.01	0.41
P_1_	45	44.31	56.55	4.23	0.57	0.32
P_2_	68	91.13	72.18	0.16	0.13	0.11
P_3_	223	94.55	73.22	0.039	0.023	0.042
P_4_	905	97.03	73.59	0.015	0.017	0.039

CCS: Cold compressive strength.

**Table 6:** Compositions of the zinc-bearing dust pellets before reduction (EDS analysis, points on Figure 7, atomic conc, %).

Point No.	Elements										Possible Phases
	Fe	O	Si	Ca	Mg	Al	Zn	K	Na	C	
1	100	0	0	0	0	0	0	0	0	0	Fe
2	41.3	58.7	0	0	0	0	0	0	0	0	Fe_3_O_4_
3	40.5	59.5	0	0	0	0	0	0	0	0	Fe_2_O_3_
4	29.3	56.9	0	13.8	0	0	0	0	0	0	CaFe_2_O_4_
5	29.7	57.7	0	0	12.6	0	0	0	0	0	MgFe_2_O_4_
6	0	67.9	32.1	0	0	0	0	0	0	0	SiO_2_
7	0	61.6	0	0	0	38.4	0	0	0	0	Al_2_O_3_
8	0	0	0	0	0	0	0	0	0	100	C
9	29.8	58.1	0	0	0	0	12.1	0	0	0	ZnFe_2_O_4_
10	40.2	59.8	0	0	0	0	0	0	0	0	Fe_2_O_3_
11	36.7	57.7	0	0	0	0	0	0	5.6	0	NaFeO_2_, Fe_2_O_3_
12	37.1	56.5	0	0	0	0	0	6.4	0	0	KFeO_2_, Fe_2_O_3_

**Table 7:** Compositions of the zinc-bearing dust pellets after reduction (EDS analysis, points on Figure 5, atomic conc, %).

Point No.	Elements									Possible Phases
	Fe	O	Si	Ca	Mg	Al	Zn	K	Na	
1	100	0	0	0	0	0	0	0	0	Fe
2	51.2	48.8	0	0	0	0	0	0	0	FeO
3	45.8	43.9	0	0	0	0	10.3	0	0	ZnFe_2_O_4_, Fe
4	85.4	11.1	0	0	0	0	0	0	3.5	NaFeO_2_, Fe
5	88.9	8.8	0	0	0	0	0	2.3	0	KFeO_2_, Fe
6	0	60.4	24.3	8.7	6.6	0	0	0	0	CaMgSi_2_O_6_
7	91.3	7.3	0	0	0	0	1.4	0	0	ZnFe_2_O_4_, Fe
8	93.6	4.8	0	0	0	0	0	0	1.6	NaFeO_2_, Fe
9	96.3	3.1	0	0	0	0	0	0.6	0	KFeO_2_, Fe
10	0	62.0	8.4	15.9	0	13.7	0	0	0	Ca_2_Al_2_SiO_7_
11	0	60.5	21.1	10.5	7.9	0	0	0	0	CaMgSi_2_O_6_
12	35.2	52.2	0	0	12.6	0	0	0	0	MgFe_2_O_4_
13	0	57.8	17.3	16.3	0	8.6	0	0	0	Ca_3_Al_2_Si_3_O_12_

**Table 8:** Surface areas and porosities of the PCs.

Samples	Surface Area (m^2^/g)	Total Pore Volume (mL/g)	Micropore (<2 nm)		Mesopore	
			Volume (mL/g)	Percentage (%)	Volume (mL/g)	Percentage
						(%)
PC_1_	218.9	0.191	0.100	52.37	0.091	47.63
PC_2_	314.8	0.273	0.135	49.63	0.138	50.37
PC_3_	300.65	0.264	0.127	48.21	0.137	51.79

**Table 9:** The main reactions present in the reduction and activation process.

NO.	Reaction Types	Reactions
(1)	Activation reaction, Boudouard reaction	C + CO_2_ → 2CO
(2)	Reduction reactions of iron-bearing phases	3Fe_2_O_3_ + CO → 2Fe_3_O_4_ + CO_2_
(3)		Fe_3_O_4_ + CO → 3FeO + CO_2_
(4)		FeO + CO → Fe + CO_2_
(5)		CaFe_2_O_4_ + 3CO → CaO + 2Fe + 3CO_2_
(6)		MgFe_2_O_4_ + 3CO → MgO + 2Fe + 3CO_2_
(7)	Reduction reactions of harmful metal oxides	ZnFe_2_O_4_ + 4CO → Zn + 2Fe + 4CO_2_
(8)		KFeO_2_ + 2CO → Fe + 2CO_2_ + K
(9)		NaFeO_2_ + 2CO → Fe + 2CO_2_ + Na
(10)	Reactions of gangue phases	2CaO + SiO_2_ + Al_2_O_3_ → Ca_2_Al_2_SiO_7_
(11)		CaO + 2SiO_2_ + MgO → CaMgSi_2_O_6_
(12)		3CaO + 3SiO_2_ + Al_2_O_3_ → Ca_3_Al_2_Si_3_O_12_

## Data Availability

The data presented in this study are available on request from the corresponding author. The data are not publicly available due to institutional policies that restrict data sharing.
